# Implantable cardiac defibrillator outcomes in octogenarians

**DOI:** 10.1002/joa3.70039

**Published:** 2025-03-11

**Authors:** Muhammad Rehan Zahid, Syed Tawassul Hassan, Muhammad Shaheer Bin Faheem, Aleeza Rehman, Syed Muhammad Ali

**Affiliations:** ^1^ Sialkot Medical College Madharian Wala Kalar Punjab Pakistan; ^2^ Liaquat University of Medical and Health Sciences Jamshoro Sindh Pakistan; ^3^ Department of Medicine and Surgery Karachi Institute of Medical Sciences, KIMS Karachi Pakistan; ^4^ CMH Institute of Medical Sciences CIMS Multan Pakistan; ^5^ Hamad Medical Corporation Doha Qatar

Dear Editor,

We read the article “Implantable cardiac defibrillator outcomes in octogenarians” and appreciate the authors, Stringer et al., for their efforts in examining the outcomes of implantable cardiac defibrillator (ICD) in overlooked elderly population (80 or <) having increased susceptibility to sudden cardiac death (SCD).^1^ We highly commend their work for analyzing outcomes like mortality, frailty, and usage frequency related to ICD treatment and acknowledge their contribution to the ongoing discussion of ICD therapy in older adults. However, we found several methodological gaps that can significantly affect this study's findings.

Firstly, the study relied on univariate statistical tests without any multivariate adjustments. This can fail to provide the accurate relationship of covariates such as demographics that include age and gender and comorbidities like hypertension, diabetes, and atrial fibrillation with the study outcomes, excluding necessary confounders that can significantly impact the effect of ICD and study conclusions.^2^ Although the Dalhousie frailty score helps in risk stratification and better frailty assessment in octogenarians, it requires detailed data on patients' health conditions, while the study documented a significant loss of device records and follow‐up data that can lead to inaccurate findings. Further, it requires subjective clinical judgment rather than relying on medical records, as in this study. Authors had assumed the patients to be alive who had a clinical or emergency visit in the last 3 months of the study, inducing misclassification bias as the patients who were not able to report due to any reason might be classified as dead, distorting mortality outcomes. Among patients in the primary prevention group, a set threshold of 188 bpm for ventricular tachycardia (VT)/Ventricular fibrillation (VF) is not suitable for those who experience severe arrhythmias at lower cardiac rates, and a nonindividualized programming approach can cause excessive shocks affecting the quality of life of these patients.^3^ Also, aggressive ATP usage before shocks can delay the needed shocks for patients with prolonged ventricular arrhythmias.^3^ A total of five patients (6.3%) were classified as critically frail; this underpowered subgroup analysis can increase the risk of type 2 errors, undermining the outcomes of this study.^4^ Women have a higher risk of procedural and postimplantation ICD‐related complications, but only 14% of the total population of this study were female, limiting the generalizability of findings toward the female population.^5^

Lastly, to control confounders, we recommend using multivariable regressions and adapting the electronic frailty index designed for retrospective study designs to enhance the data's reliability. However, misclassification bias can be avoided by linking records with national databases for death registries, and the ICD programming protocol should be individualized to improve patient outcomes. Further, upscaling sample sizes of subgroups and demographics would increase the statistical power and generalizability of the study.

## CONFLICT OF INTEREST STATEMENT

None.

## Data Availability

No new data are generated.
